# The longitudinal course of pediatric acute respiratory distress syndrome and its time to resolution: A prospective observational study

**DOI:** 10.3389/fped.2022.993175

**Published:** 2022-11-22

**Authors:** Judith Ju Ming Wong, Herng Lee Tan, Rehena Sultana, Yi-Jyun Ma, Apollo Aguilan, Siew Wah Lee, Pavanish Kumar, Yee Hui Mok, Jan Hau Lee

**Affiliations:** ^1^ Children's Intensive Care Unit, Department of Pediatric Subspecialties, KK Women's and Children's Hospital, Singapore, Singapore; ^2^ Duke-NUS Medical School, Singapore, Singapore; ^3^ Center for Quantitative Medicine, Duke-NUS Medical School, Singapore, Singapore; ^4^ Pediatric Intensive Care Unit, Hospital Tengku Ampuan Rahimah, Selangor, Malaysia; ^5^ Translational Immunology Institute, SingHealth/Duke-NUS Academic Medical Centre, Singapore, Singapore

**Keywords:** non-invasive ventilation, oxygen inhalation therapy, acute lung injury, acute respiratory distress syndrome, pediatric intensive care unit, critical care outcomes, artificial respiration

## Abstract

**Background:**

The longitudinal course of patients with pediatric acute respiratory distress syndrome (PARDS) is not well described. In this study, we describe the oxygenation index (OI) and oxygen saturation index (OSI) in mild, moderate, and severe PARDS over 28 days and provide pilot data for the time to resolution of PARDS (*T*_res_), as a short-term respiratory-specific outcome, hypothesizing that it is associated with the severity of PARDS and clinical outcomes.

**Methods:**

This prospective observational study recruited consecutive patients with PARDS. OI and OSI were trended daily over 28 days. *T*_res_ (defined as OI < 4 or OSI < 5.3 on 2 consecutive days) were described based on PARDS severity and analyzed with Poisson and logistic regression to determine its association with conventional outcomes [mechanical ventilation (MV) duration, intensive care unit (ICU) and hospital length of stay, 28-day ventilator-free days (VFD), and 28-day ICU-free days (IFD)].

**Results:**

There were 121 children included in this study, 33/121(27.3%), 44/121(36.4%), and 44/121(36.4%) in the mild, moderate, and severe groups of PARDS, respectively. OI and OSI clearly differentiated mild, moderate, and severe groups in the first 7days of PARDS; however, this differentiation was no longer present after 7days. Median *T*_res_ was 4 (interquartile range: 3, 6), 5 (4, 7), and 7.5 (7, 11.5) days; *p* < 0.001 for the mild, moderate, and severe groups of PARDS, respectively. *T*_res_ was associated with increased MV duration, ICU and hospital length of stay, and decreased VFD and IFD.

**Conclusion:**

The oxygenation defect in PARDS took progressively longer to resolve across the mild, moderate, and severe groups. *T*_res_ is a potential short-term respiratory-specific outcome, which may be useful in addition to conventional clinical outcomes but needs further validation in external cohorts.

## Introduction

Pediatric acute respiratory distress syndrome (PARDS) is characterized by severe hypoxemia (1). Clinical definitions of PARDS (or ARDS) invariably incorporate some measure of oxygenation [e.g., oxygenation index (OI), oxygen saturation index (OSI), partial pressure of arterial oxygen to fraction of inspired oxygen (PF) ratio, and oxygen saturation to the fraction of inspired oxygen (SF) ratio] with cut-offs delineating mild, moderate, and severe groups (1, 2). However, most conventional outcomes [e.g., mortality, ventilator duration, and pediatric intensive care unit (PICU) duration] in PARDS are often affected by confounders (3) and are frequently not due to refractory hypoxemia (4, 5). There is a lack of a more direct and specific respiratory outcome for PARDS. Oxygenation measures are associated with outcomes and the ability to stratify patients into prognostic groups (6, 7). As such, it is intuitive that the resolution of this oxygenation defect may result in a positive short-term respiratory-specific outcome (8). In adult patients, the presence or absence of resolution of ARDS (defined as improvement in *P*/*F* > 200 for at least 48 h) was shown to be associated with lower hospital mortality (8).

Most previous studies in PARDS focused on the first few days of illness and rarely examined the course of illness to its resolution in detail (6, 9). Indeed, PARDS may progress in severity after diagnosis and this trajectory may be associated with worse outcomes (10). It is also possible that patients with PARDS are vulnerable to further respiratory insult necessitating escalation of respiratory support. The understanding of the course/trajectory of PARDS is lacking and is an unmet medical need. To address these gaps in the medical literature, we undertook this study with the aims of (1) describing the extent and longitudinal course of lung injury in mild, moderate, and severe PARDS by ascertaining the OI and OSI trends over 28 days and (2) demonstrating proof of concept of time to resolution of PARDS (*T*_res_) as a short-term respiratory-specific outcome. We hypothesized that *T*_res_ is associated with the severity of PARDS and clinical outcomes.

## Methods

### Design, setting, and patients

This study was conducted in a 16-bedded multidisciplinary PICU from September 2018 to July 2021. All PICU admissions were screened daily for PARDS and informed consent was obtained under the centralized Singhealth institutional review board reference number: CIRB 3076/2017/E. The Pediatric Acute Lung Injury Consensus Conference (PALICC) criteria were applied to identify patients with PARDS and oxygenation criteria were met on two separate blood gases 4 h apart (11, 12). All patients were ventilated according to a lung-protective mechanical ventilation protocol (13). Reporting was in accordance with the Strengthening the Reporting of Observational Studies in Epidemiology (STROBE) guidelines (Supplementary appendix). (14).

### Measurement and data collection

Clinical data were collected which included admission severity scores [Pediatric Index of mortality (PIM) 2 and Pediatric Logistic Organ Dysfunction (PELOD) scores] (15, 16). Comorbidities were defined by the presence of complex chronic conditions and categorized into the most clinically affected system (17). Sepsis and organ dysfunction were defined by the International Pediatric Sepsis Consensus Conference (18). Pediatric Overall Performance Category (POPC) and Pediatric Cerebral Performance Category (PCPC) were scored at PICU admission and discharge (19). Mechanical ventilation (MV) settings and their corresponding blood gas measurements were recorded at 0600-0800H daily—these were used for calculation of daily OI and OSI up to 28 days after diagnosis PARDS.

### Outcomes and statistical analysis

Patients were analyzed in three groups: mild, moderate, and severe PARDS. The highest severity over the first 7 days of PARDS was used to categorize patients into their severity groups (e.g., if a patient was recruited on day 1 with mild PARDS but progressed to develop severe PARDS on day 3, he/she was analyzed as severe PARDS). This was done to capture all patients who developed severe disease who will likely have poorer outcomes compared to patients who remain in the mild/moderate category throughout their illness (10). Patients who remained on non-invasive ventilation throughout the course of PARDS were empirically categorized into the mild group. Data were summarized as counts (percentages) and median (interquartile range) for categorical and continuous variables, respectively. Comparisons between severity groups were done using the Chi-square test and Kruskal–Wallis tests for categorical and continuous variables, respectively.

The primary outcome was time to resolution of PARDS (*T*_res_) defined as OI < 4 or OSI < 5.3 for two consecutive days—this was treated as time-to-event data. *T*_res_ based on PARDS severity was plotted using a Kaplan–Meier curve and compared using the Log-rank test. We also established the relationship between *T*_res_ with PARDS severity, PIM2, and PELOD scores using Cox regression to determine if the general severity of illness impacts the resolution of PARDS. *T*_res_ (treated as a continuous variable) was further analyzed to quantify its association with conventional PARDS outcomes using Poisson [for ICU length of stay, hospital length of stay, 28-intensive care unit free days (IFD), duration of MV, 28-ventilator-free days (VFD)] and Cox regression (for change in POPC and PCPC from admission discharge). These associations were expressed as an incidence rate ratio (IRR), hazard ratio (HR), or odds ratio (OR), whichever is appropriate, with corresponding 95% confidence intervals (CI). After a review of the causes of death, a sensitivity analysis was performed excluding patients who at PICU admission, had a poor overall diagnosis (e.g., terminal malignancy) or poor neurologic prognosis (e.g., brainstem dysfunction). Both survivors and non-survivors were included in the analysis of *T*_res_ with censoring of non-survivors at the time of death. A sensitivity analysis for the primary outcome (*T*_res_) was done using conventional stratification of PARDS within 24 h of diagnosis.

Analysis was performed on STATA software, version 15.1 (StataCorp, College Station, TX) and SAS version 9.3 software (SAS Institute, Cary, NC). All tests were two-tailed and a *p*-value <0.05 was accepted as statistically significant.

## Results

One hundred and twenty-one patients were identified for this study, with 33/121 (27.3%, 44/121 (36.4%), and 44/121 (36.4%) in the mild, moderate, and severe groups, respectively. The majority of patients in this cohort had pneumonia [78/121 (64.5%)] as the inciting factor for PARDS followed by sepsis [20/121 (12.4%)] (Table 1). Most patients had underlying comorbidities [85/121 (70.3%)], of which neuromuscular [29/121 (27.1%)] and genetic/congenital [21/121 (19.6%)] were the most common.

**Table 1:** Characteristics of patients with pediatric acute respiratory distress syndrome.

Characteristics	Mild PARDS (*n* = 33)	Moderate PARDS (*n* = 44)	Severe PARDS (*n* = 44)	Total (*n* = 121)	*p-*Value
Age, years	4.5 (0.8, 12.6)	3.3 (0.6, 9.4)	1.5 (0.5, 5.1)	2.8 (0.6, 9.4)	0.227
Male gender	19 (57.6)	25 (56.8)	31 (70.5)	75 (62.0)	0.348
Weight, kg	13.9 (8.6, 32.3)	12.6 (6.7, 30.2)	9.7 (6.2, 14.3)	12 (6.9, 29.2)	0.183
BMI, kg/m^2^	16.5 (13.6, 20.0)	16.8 (13.7, 19.1)	16.9 (15.2, 19.2)	16.7 (13.8, 19.3)	0.996
Comorbidity					0.328
Neuromuscular	6 (19.4)	14 (37.8)	9 (23.1)	29 (27.1)	
Respiratory	2 (6.5)	2 (5.4)	4 (10.3)	8 (7.5)	
Heme-oncology	2 (6.5)	3 (8.1)	5 (12.8)	10 (9.4)	
Genetic/congenital	5 (16.1)	10 (27.0)	6 (15.4)	21 (19.6)	
Neoplastic	2 (6.5)	1 (2.7)	4 (10.3)	7 (6.5)	
Others	3 (9.7)	4 (10.8)	3 (7.7)	10 (9.4)	
PIM 2	5.5 (2.8, 22.2)	6.2 (3.2, 15.8)	11.3 (4.4, 24.3)	7.1 (3.3, 20.9)	0.269
PELOD	9 (1, 11)	2 (1, 14)	10 (1.5, 20)	10 (1, 13.5)	0.489
Risk factor					
Pneumonia	21 (63.6)	26 (59.1)	31 (70.5)	78 (64.5)	0.422
Aspiration	3 (9.1)	7 (15.9)	4 (9.1)	14 (11.6)	
Sepsis	4 (12.1)	9 (20.5)	7 (15.9)	20 (16.5)	
Others	5 (15.2)	2 (4.6)	2 (4.6)	9 (7.4)	
Bacteremia	2 (6.1)	6 (13.6)	7 (15.9)	15 (12.4)	0.410
Respiratory pathogen					
Bacterial	10 (30.3)	14 (31.8)	21 (47.2)	45 (37.2)	0.192
Viral	15 (45.5)	22 (50.0)	24 (54.6)	61 (50.4)	0.730
Fungal	2 (6.1)	3 (6.8)	9 (20.5)	14 (11.6)	0.069
None	8 (24.2)	8 (18.2)	9 (20.5)	25 (20.7)	0.809
Air leak	1 (3.0)	2 (4.6)	5 (11.4)	8 (6.6)	0.273
Multiorgan dysfunction	18 (56.3)	27 (61.4)	36 (81.8)	81 (67.5)	0.035
POPC admission	1.5 (1, 4)	3 (1, 4)	2 (1, 4)	2 (1, 4)	0.297
POPC discharge	4 (1, 4)	4 (4, 4)	4 (4, 4)	4 (3, 4)	0.253
PCPC admission	1.5 (1, 4)	3 (2, 4)	2 (1, 4)	2 (1, 4)	0.166
PCPC discharge	3 (1, 4)	4 (3, 4)	4 (3,4)	4 (3,4)	0.046

Continuous and categorical variables summarized in medians (interquartile ranges) and counts (percentages), respectively.

BMI, body mass index; PIM 2, Pediatric Index of Mortality 2; PELOD, pediatric logistic organ dysfunction; PARDS, pediatric acute respiratory distress syndrome; POPC, pediatric overall performance category; PCPC, pediatric cerebral performance category.

**Figure 1:**
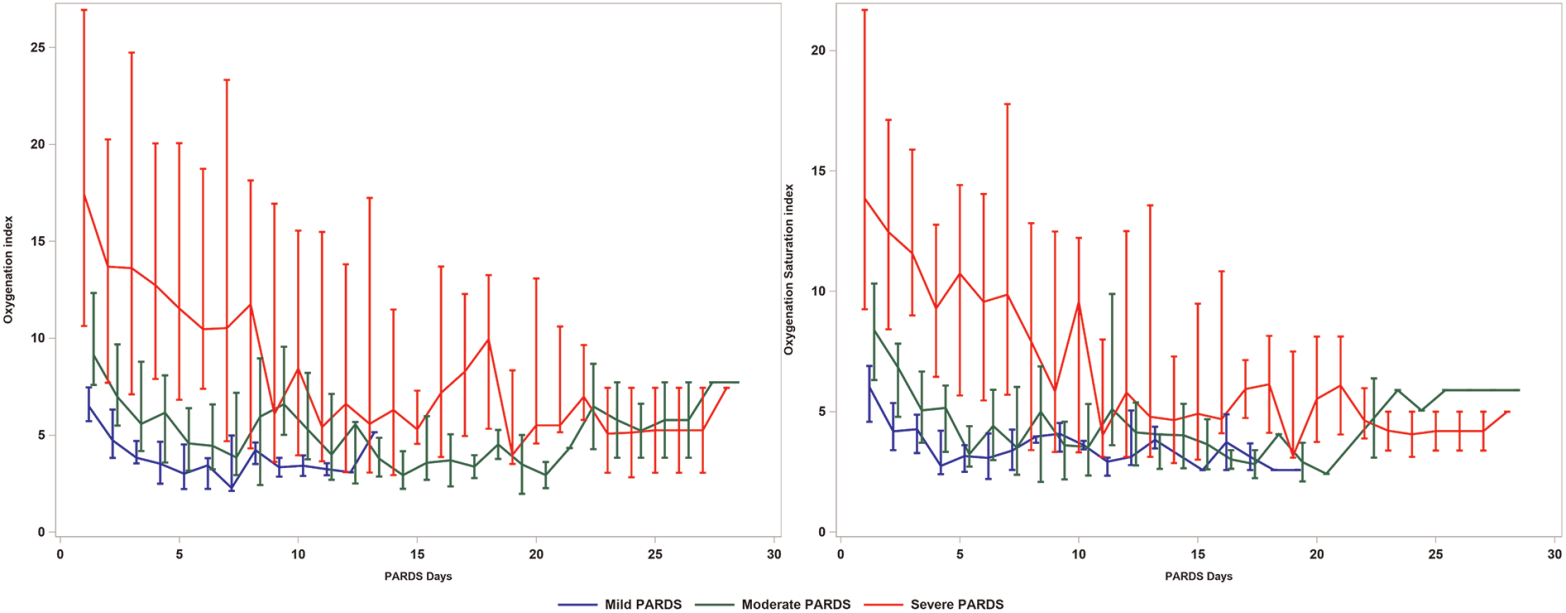
Oxygenation index and oxygenation saturation index in patients with PARDS across 28 days. PARDS, pediatric acute respiratory distress syndrome.

Almost all patients [115/121 (95.0%)] required invasive MV (Supplementary Figure S1). Throughout the first 7 days of PARDS, the OI, OSI and alveolar-arterial oxygen gradient (AaDO2) were higher and PF ratio, SF ratio were lower with greater severity of PARDS (Supplementary Table S1). Differences in OI and OSI (as well as the other oxygenation measures) were not statistically significant after the first week of PARDS (Figure 1). There was a stepwise increase in the use of pulmonary (e.g., high-frequency oscillation, pulmonary vasodilators, prone positioning) and non-pulmonary (e.g., neuromuscular blockade, diuretics, and red blood cell transfusions) therapies, across severity groups (Supplementary Table S2). A stepwise increase in the vasoactive inotrope scores and other PICU support therapies was also evident across severity groups (Supplementary Table S3).

MV lasted longer and VFD lasted shorter across severity groups (Table 2). Patients with severe PARDS required more time to be liberated from the ventilator (Log-rank test *p* = 0.0294), whereas there was no difference between the mild and moderate groups (Supplementary Figure S2). Change in the PCPC score from admission to discharge was higher in severe PARDS compared to moderate or mild PARDS [(1 (0, 2) vs. 0 (0, 1) and 0 (0, 1), respectively; *p* = 0.045], but there was no significant difference for the change of POPC score. PICU duration was longer and IFD shorter across severity groups. Time to PICU discharge was successively longer with increasing PARDS severity (Log-rank test *p* = 0.0003) (Supplementary Figure S3). Hospital duration was increased across severity groups and the Kaplan–Meier plot showed that the time to hospital discharge was different across severity groups (Log-rank test *p* = 0.0008) (Supplementary Figure S4). There was no difference in PICU and hospital mortality across severity groups (Supplementary Figures S5, 6). Death due to refractory hypoxemia occurred only in the severe group, whereas, deaths due to multiorgan dysfunction occurred in all groups (Supplementary Table S4).

**Table 2:** Short and intermediate term outcomes in patients with pediatric acute respiratory distress syndrome.

Outcomes	Mild PARDS (*n* = 33)	Moderate PARDS (*n* = 44)	Severe PARDS (*n* = 44)	Total (*n* = 121)	*p-*Value
Time to resolution of PARDS, days	4 (3, 6)	5 (4, 7)	7.5 (7, 11.5)	6 (4, 8)	<0.001
Ventilator duration, days	5 (3, 10)	7 (4, 13)	11.5 (8, 19.5)	8 (4, 13)	0.001
28-day VFD	21.5 (0, 24)	21 (12, 23.5)	13 (0, 18)	17 (0, 22)	0.004
PICU mortality	6 (18.2)	4 (9.1)	8 (18.2)	18 (14.9)	0.401
PICU duration, days	5.5 (3.5, 10.5)	11 (6, 16)	15 (9.5, 30)	11 (6, 19)	<0.001
28-day IFD	21 (0, 24)	16 (0, 21)	2.5 (0, 13.5)	14 (0, 21)	<0.001
Hospital duration, days	16 (6, 24)	27 (17, 60)	31 (17.5, 69)	23 (12, 56)	0.007
Hospital mortality	7 (21.2)	4 (9.1)	10 (22.7)	21 (17.4)	0.190

Continuous and categorical variables summarized in medians (interquartile ranges) and counts (percentages), respectively.

IFD, PICU free days; PARDS, pediatric acute respiratory distress syndrome; PICU, pediatric intensive care unit; VFD, ventilator free days.

**Figure 2:**
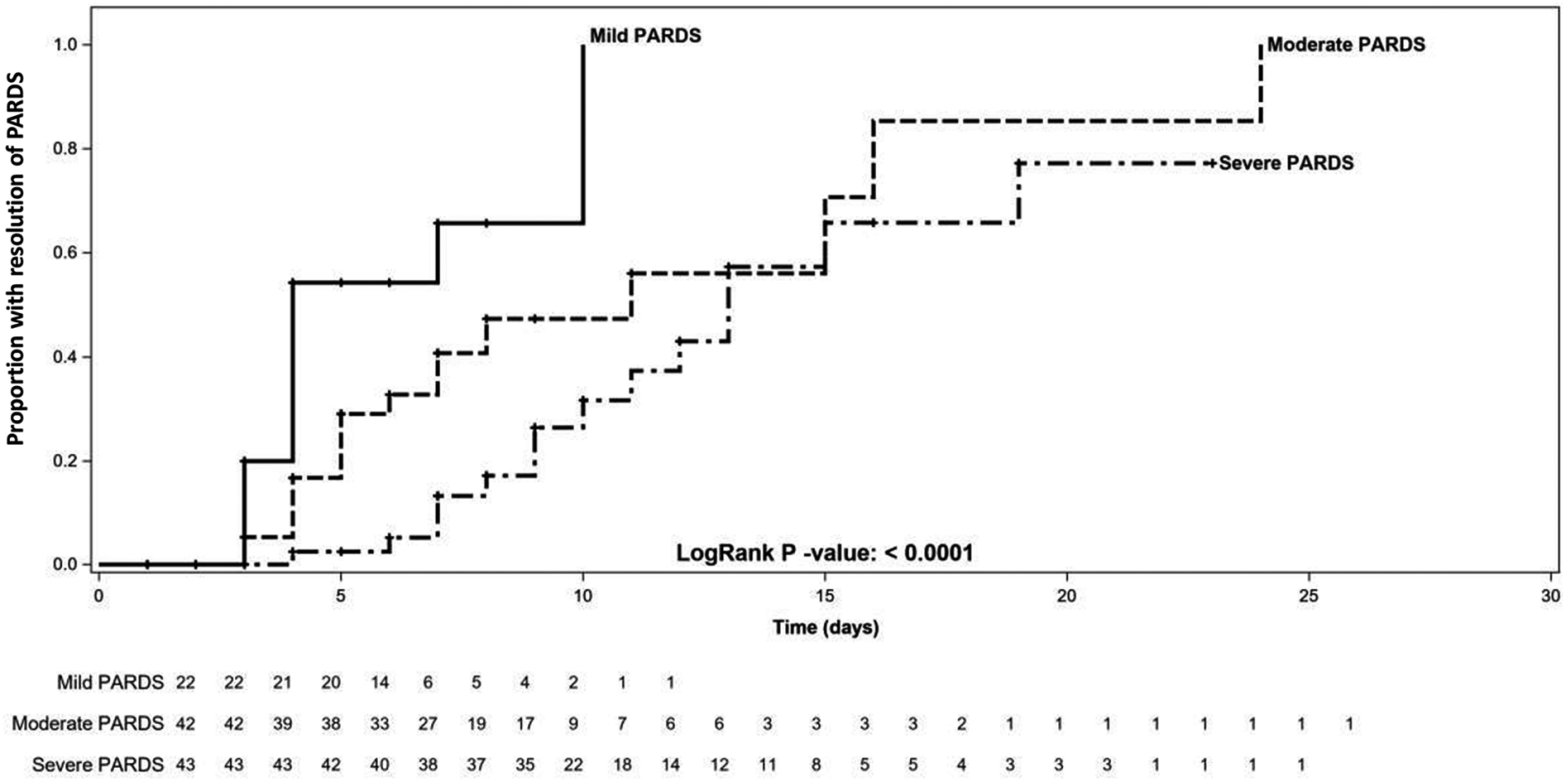
Kaplan–Meier curve of time to resolution of pediatric acute respiratory distress syndrome in patients with mild, moderate, and severe disease. PARDS, pediatric acute respiratory distress syndrome.

The median (interquartile range) *T*_res_ demonstrated a stepwise increase from the mild to severe PARDS categories [mild 4 (3, 6), moderate 5 (4, 7) vs. severe 7.5 (7, 11.5) days; *p* < 0.0001] (Table 2 and Figure 2). There was a decreased likelihood of PARDS resolution in moderate [HR 0.35 (95%CI 0.16, 0.76); *p* = 0.008] and severe [HR 0.17 (95%CI 0.08, 0.39); *p* < 0.001] PARDS compared to mild PARDS. However, there was no association between PIM 2 [HR 1.01 (95%CI 0.99, 1.02); *p* = 0.253] and PELOD [HR 1.02 (95%CI 0.99, 1.05); *p* = 0.326] scores and *T*_res_. *T*_res_ was associated with an increased duration of MV [IRR 1.10 (95%CI 1.05, 1.15); *p* < 0.001], PICU length of stay [IRR 1.11 (95%CI 1.06, 1.16); *p* < 0.001], and hospital length of stay [IRR 1.06 (95%CI 1.01, 1.11); *p* = 0.018]. In addition, *T*_res_ was associated with decreased VFDs [IRR 0.93 (95%CI 0.87, 1.00); *p* = 0.046] and IFDs [IRR 0.84 (95%CI 0.76, 0.92); *p* < 0.001]. There was no association between *T*_res_ and the change in POPC [HR 1.05 (95%CI 0.83, 1.33); *p* = 0.688] and PCPC [HR 1.13 (95%CI 0.84, 1.52); *p* = 0.424] scores. The sensitivity analysis for the primary outcome (*T*_res_) according to severity classification within 24 h of diagnosis showed a similar direction of effect.

## Discussion

Our study described OI and OSI trends in patients with PARDS over the course of 28 days, as well as the time to resolution. OI and OSI provided a clear separation between mild, moderate, and severe groups in the first week of PARDS. We demonstrated pilot data that *T*_res_ lengthened with increasing severity of PARDS and that the likelihood of PARDS resolution was lower in moderate and severe PARDS compared with mild PARDS. In contrast, increased overall severity of illness (PIM2 and PELOD) was not associated with a lower likelihood of PARDS resolution. We went on to demonstrate that a longer *T*_res_ was associated with clinical outcomes such as duration of MV and length of stay.

We examine in detail the longitudinal course of PARDS with respect to the oxygenation trends. This is important because patients with mild/moderate PARDS may progress to severe PARDS after the first 24 h (20). Patients who progress to severe PARDS, even if this occurs days later, may benefit from therapies for severe PARDS and may have similar poorer intermediate/long-term outcomes (e.g., mortality, MV duration, or long-term respiratory support) as severe PARDS. There are data in adult ARDS that approximately 20% of patients progress in severity and this may be associated with poorer prognosis (10). Future studies are needed to compare the outcomes of patients who progress in severity vs. those who remain in their severity groups or resolve, in order to confirm whether these patients actually perform worse. The discovery that oxygenation measures were useful in disease stratification within the first 7 days of PARDS suggests that this critical period should be minimally included in future PARDS studies. It is unclear why there was such poor differentiation in OI/ OSI between the severity groups after 7 days, but it could be due to the smaller number of patients who remained intubated and had data for OI/OSI calculation.

There is currently no physiologic marker that indicates recovery from lung injury. Here, the oxygenation defect that characterizes PARDS, which was demonstrated to improve with time, could be used as a respiratory-specific outcome corresponding to the physiologic recovery of lung injury. We demonstrated that *T*_res_ was specifically associated with the severity of PARDS but not with the general severity of illness (PIM2 and PELOD scores) and how it related to other clinical outcomes. We highlight the bias by using conventional outcomes (Supplementary Figure S7). Conventional outcomes are confounded by patient factors (e.g., pneumonia is a common terminal event in end-stage malignancies and will inevitably be associated with poor survival outcomes) and therapeutic factors (e.g., use of prolonged neuromuscular blockade and systemic corticosteroids for any indication may result in adverse functional outcomes independent of PARDS course), many of which are not respiratory in nature (3). Indeed, mortality due to refractory hypoxemia accounts for only approximately 20% of deaths in pediatric and adult cohorts of ARDS (i.e., 80% of patients with ARDS die from other causes) (4, 5, 21, 22)—this would also confound other outcome measures where mortality was included as part of that composite outcome (e.g., VFD, IFD and PARDS-free days). Whereas, the duration of MV may be confounded by non-pulmonary disease (e.g., a patient with PARDS who has underlying neurological comorbidity may remain on ventilation long after resolution of lung disease). It is evident from Supplementary Figure S7 that many patients remain intubated/admitted to the PICU for days/weeks after the resolution of PARDS, presumably due to factors other than acute lung injury. *T*_res_, therefore, may be useful in addition to conventional patient-important outcomes when studying PARDS specific therapies.

There are limitations to this study. Despite routine and complete screening of all PICU admissions, our cohort had a small sample size. The single-center nature of this study also limits its generalizability. A future multicenter study will address both these limitations. Because this study is only proof of concept, a separate study with larger and independent cohort of PARDS patients is needed to validate *T*_res_ as a useful clinical and research outcome measure. From our data, a post hoc sample size calculation to detect a difference in *T*_res_ of 3.5 days between severe and non-severe PARDS will require a sample size of 44 and 77, respectively (based on the following parameters: recruitment period of 36 months, follow-up for 28 days, allocation ratio severe to non-severe 1:1.5, alpha 5%, power 80%). Another limitation was that we did not evaluate the relationship between *T*_res_ and longer-term outcomes in PARDS, e.g., duration of non-invasive respiratory support or follow-up lung function—this should be evaluated in future studies.

## Conclusion

The oxygenation defect associated with PARDS was demonstrated to subside towards the end of the first week of illness, with severe disease taking longer to resolve than mild or moderate disease. We propose *T*_res_ as a surrogate outcome measure for PARDS (specifically indicating resolution of the oxygenation defect occurring in PARDS), in addition to conventional outcomes like mortality and duration of MV which are less specific for PARDS. External validation of these findings in a larger and independent cohort is necessary to evaluate *T*_res_ as a relevant clinical outcome measure.

## Data Availability

The raw data supporting the conclusions of this article will be made available by the authors, without undue reservation.
