# Supplementary material for: The longitudinal course of pediatric acute respiratory distress syndrome and its time to resolution: A prospective observational study
Source: Front Pediatr. 2022 Nov 22;10:993175. doi: 10.3389/fped.2022.993175 (PMC9723458; doi:10.3389/fped.2022.993175)
Supplement: Supplementary file 1 [file Datasheet1.docx]

**Supplementary Material**

**Contents**

| No. | Table / Figure | Page |
| --- | --- | --- |
| 1 | eTable 1: Oxygenation indices over the first seven days in patients with pediatric acute respiratory distress syndrome | 2 |
| 2 | eTable 2: Pulmonary and non-pulmonary therapies in patients with pediatric acute respiratory distress syndrome | 3 |
| 3 | eTable 3: Daily vasoactive inotrope score and other intensive care unit support therapies in patients with pediatric acute respiratory distress syndrome | 4 |
| 4 | eTable 4: Cause of death in patients with pediatric acute respiratory distress syndrome | 5 |
| 5 | eFigure 1: Ventilation parameters in patients with PARDS across 28 days | 6 |
| 6 | eFigure 2: Kaplan-Meier curves of duration of mechanical ventilation in patients with mild, moderate and severe pediatric acute respiratory distress syndrome | 7 |
| 7 | eFigure 3: Kaplan-Meier curves of PICU length of stay in patients with mild, moderate and severe pediatric acute respiratory distress syndrome | 8 |
| 8 | eFigure 4: Kaplan-Meier curves of hospital length of stay in patients with mild, moderate and severe pediatric acute respiratory distress syndrome | 9 |
| 9 | eFigure 5: Kaplan-Meier curve for outcome of intensive care unit mortality in patients with mild, moderate and severe pediatric acute respiratory distress syndrome and sensitivity analysis excluding patients who at admission, had a poor overall or poor neurologic prognosis | 10 |
| 10 | eFigure 6: Kaplan-Meier curve for outcome of hospital mortality in patients with mild, moderate and severe pediatric acute respiratory distress syndrome and sensitivity analysis excluding patients who at admission, had a poor overall or poor neurologic prognosis | 11 |
| 11 | eFigure 7: Timeline of time to resolution of pediatric acute respiratory distress syndrome and discharge from the intensive care unit | 12 |

**eTable 1: Oxygenation indices over the first seven days in patients with pediatric acute respiratory distress syndrome**

| **Oxygenation** | **Mild PARDS (n=33)** | **Moderate PARDS (n=44)** | **Severe PARDS (n=44)** | **Total**  **(n=121)** | **P value** |
| --- | --- | --- | --- | --- | --- |
| Oxygenation index | | | | | |
| Day 1 | 6.2 (5.5, 7.2) | 10.3 (8.5, 12.3) | 16.6 (9.8, 25.8) | 9.9 (7.2, 15.0) | <0.001 |
| Day 2 | 4.7 (3.8, 6.3) | 7.0 (5.5, 9.7) | 13.7 (7.7, 20.3) | 7.4 (5.2, 13.5) | <0.001 |
| Day 3 | 3.8 (3.5, 4.7) | 5.6 (4.2, 8.8) | 13.6 (7.1, 24.7) | 6.5 (4.2, 13.3) | <0.001 |
| Day 4 | 3.5 (2.5, 4.7) | 6.2 (3.6, 8.1) | 12.4 (7.9, 20.1) | 7.2 (3.9, 11.9) | <0.001 |
| Day 5 | 3.0 (2.2, 4.5) | 4.6 (3.2, 6.4) | 11.5 (6.8, 20.1) | 5.9 (3.6, 11.8) | <0.001 |
| Day 6 | 3.4 (2.2, 5.1) | 4.4 (3.2, 6.6) | 10.7 (7.7, 20.2) | 6.6 (3.7, 10.8) | <0.001 |
| Day 7 | 2.3 (2.1, 5.0) | 4.5 (3.1, 8.2) | 9.9 (4.4, 23.3) | 5.6 (3.2, 11.5) | <0.001 |
| Oxygenation saturation index | | | | | |
| Day 1 | 6.0 (4.7, 8.0) | 9.6 (7.1, 10.9) | 12.1 (7.5, 18.1) | 9.1 (6.5, 12.6) | <0.001 |
| Day 2 | 4.2 (3.4, 5.4) | 6.2 (4.8, 7.8) | 10.1 (6.7, 16.1) | 6.5 (4.8, 9.6) | <0.001 |
| Day 3 | 4.1 (3.3, 4.4) | 4.4 (3.3, 6.6) | 10.5 (5.7, 15.9) | 5.1 (3.8, 9.6) | <0.001 |
| Day 4 | 3.0 (2.7, 3.9) | 4.1 (3.0, 6.0) | 9.3 (5.6, 14.3) | 5.2 (3.3, 8.5) | <0.001 |
| Day 5 | 2.8 (2.5, 3.6) | 3.4 (2.8, 4.7) | 9.0 (5.5, 13.8) | 4.4 (3.1, 8.3) | <0.001 |
| Day 6 | 2.9 (2.2, 4.4) | 3.6 (2.8, 4.7) | 7.5 (4.9, 12.8) | 4.6 (3.1, 7.5) | <0.001 |
| Day 7 | 3.1 (2.2, 4.2) | 3.5 (2.3, 4.6) | 7.0 (3.8, 14.8) | 4.2 (2.7, 7.7) | <0.001 |
| Partial pressure of arterial oxygen: fraction of inspired oxygen ratio | | | | | |
| Day 1 | 219 (181, 267) | 160 (128, 179) | 95 (68, 129) | 152 (103, 199) | <0.001 |
| Day 2 | 266 (217, 315) | 202 (156, 259) | 128 (101, 204) | 195 (137, 261) | <0.001 |
| Day 3 | 310 (245, 383) | 223 (173, 298) | 121 (84, 196) | 213 (141, 303) | <0.001 |
| Day 4 | 351 (284, 364) | 246 (205, 318) | 134 (107, 188) | 211 (135, 304) | <0.001 |
| Day 5 | 365 (265, 393) | 259 (217, 316) | 148 (110, 220) | 224 (148, 311) | <0.001 |
| Day 6 | 342 (310, 398) | 250 (190, 343) | 143 (100, 224) | 208 (139, 310) | <0.001 |
| Day 7 | 307 (237, 408) | 250 (164, 314) | 171 (89, 277) | 236 (147, 314) | 0.004 |
| Oxygen saturation: fraction of inspired oxygen ratio | | | | | |
| Day 1 | 235 (184, 253) | 168 (129, 221) | 108 (89, 160) | 160 (109, 233) | <0.001 |
| Day 2 | 265 (248, 317) | 213 (192, 271) | 137 (95, 198) | 205 (172, 257) | <0.001 |
| Day 3 | 287 (238, 384) | 240 (228, 313) | 172 (119, 190) | 228 (171, 287) | <0.001 |
| Day 4 | 360 (263, 452) | 277 (243, 323) | 185 (145, 254) | 268 (190, 313) | <0.001 |
| Day 5 | 322 (269, 388) | 317 (271, 325) | 198 (144, 240) | 272 (198, 323) | <0.001 |
| Day 6 | 317 (238, 404) | 277 (228, 320) | 213 (138, 274) | 235 (194, 313) | 0.001 |
| Day 7 | 370 (273, 447) | 312 (235, 358) | 211 (113, 252) | 240 (203, 320) | <0.001 |
| Alveolar-arterial oxygen gradient | | | | | |
| Day 1 | 152 (94, 211) | 221 (186, 312) | 417 (288, 542) | 263 (175, 421) | <0.001 |
| Day 2 | 92 (53, 133) | 146 (99, 214) | 241 (173, 443) | 165 (92, 254) | <0.001 |
| Day 3 | 65 (36, 125) | 104 (73, 139) | 242 (131, 441) | 124 (70, 242) | <0.001 |
| Day 4 | 42 (26, 64) | 95 (63, 137) | 239 (129, 338) | 118 (63, 216) | <0.001 |
| Day 5 | 40 (20, 57) | 77 (40, 109) | 191 (110, 275) | 101 (49, 204) | <0.001 |
| Day 6 | 41 (17, 57) | 85 (45, 144) | 168 (116, 325) | 116 (61, 199) | <0.001 |
| Day 7 | 41 (28, 82) | 80 (44, 130) | 162 (72, 370) | 95 (50, 183) | <0.001 |

PARDS – Pediatric Acute Respiratory Distress Syndrome

**eTable 2: Pulmonary and non-pulmonary therapies in patients with pediatric acute respiratory distress syndrome**

| **Therapies** | **Mild PARDS (n=33)** | **Moderate PARDS (n=44)** | **Severe PARDS (n=44)** | **Total**  **(n=121)** | **P value** |
| --- | --- | --- | --- | --- | --- |
| HFOV | 2 (6.1) | 9 (20.5) | 31 (70.5) | 42 (34.7) | <0.001 |
| Pulmonary vasodilator | 1 (3.0) | 6 (16.4) | 19 (43.2) | 26 (21.5) | <0.001 |
| Prone position | 6 (18.2) | 7 (15.9) | 19 (43.2) | 32 (26.5) | 0.007 |
| Systemic corticosteroids | 13 (39.4) | 25 (56.8) | 26 (59.1) | 64 (52.9) | 0.186 |
| Duration, days | 4 (1, 9) | 9 (4, 23) | 10.5 (1, 20) | 5 (1, 16) | 0.103 |
| Hydrocortisone equivalent, mg/kg | 33 (9.6, 44.1) | 20.8 (13.9, 47.9) | 71.3 (25.3, 186.2) | 38.0 (15.3, 82.5) | 0.030 |
| NMB | 1 (3.0) | 8 (18.2) | 26 (59.1) | 35 (28.9) | <0.001 |
| Diuretics | 21 (63.6) | 37 (84.1) | 42 (95.5) | 100 (82.6) | 0.001 |
| RBC transfusion | 8 (24.2) | 22 (50.0) | 34 (77.3) | 64 (52.9) | <0.001 |

Continuous and categorical variables summarized in medians (interquartile ranges) and counts (percentages), respectively

HFOV – High frequency oscillatory ventilation

NMB – Neuromuscular blockade

PARDS – pediatric acute respiratory distress syndrome

RBC – red blood cell

**eTable 3: Daily vasoactive inotrope score and other intensive care unit support therapies in patients with pediatric acute respiratory distress syndrome**

| **Other PICU support** | **Mild PARDS (n=33)** | **Moderate PARDS (n=44)** | **Severe PARDS (n=44)** | **Total**  **(n=121)** | **P value** |
| --- | --- | --- | --- | --- | --- |
| Inotrope | 16 (48.5) | 28 (63.6) | 35 (79.6) | 79 (65.3) | 0.017 |
| Vasoactive inotrope score | | | | | |
| Day 1 | 0 (0, 15) | 1.5 (0, 11) | 10 (0, 20) | 1 (0, 16.5) | 0.370 |
| Day 2 | 0 (0, 5) | 3 (0, 15) | 5 (0, 27.5) | 3 (0, 15) | 0.238 |
| Day 3 | 0 (0, 0) | 0 (0, 6) | 5 (0, 19) | 0 (0, 10) | 0.040 |
| Day 4 | 0 (0, 0) | 0 (0, 4) | 1.5 (0, 8.5) | 0 (0, 5) | 0.083 |
| Day 5 | 0 (0, 0) | 0 (0, 1.5) | 0 (0, 6.5) | 0 (0, 2) | 0.019 |
| Day 6 | 0 (0, 0) | 0 (0, 0) | 0 (0, 4) | 0 (0, 0) | 0.013 |
| Day 7 | 0 (0, 0) | 0 (0, 0) | 0 (0, 3) | 0 (0, 0) | 0.033 |
| CRRT | 3 (9.1) | 5 (11.4) | 4 (9.1) | 12 (9.9) | 0.922 |
| Platelet transfusion | 6 (18.2) | 11 (25.0) | 17 (38.6) | 34 (28.1) | 0.120 |
| FFP transfusion | 6 (18.2) | 15 (34.1) | 17 (38.6) | 38 (31.4) | 0.143 |
| Cryoprecipitate transfusion | 1 (3.0) | 5 (11.4) | 10 (22.7) | 16 (13.2) | 0.037 |
| IVIG | 1 (3.0) | 5 (11.4) | 9 (20.5) | 15 (12.4) | 0.069 |
| ECMO | 0 (0.0) | 4 (9.1) | 6 (13.6) | 10 (8.3) | 0.096 |

Continuous and categorical variables summarized in medians (interquartile ranges) and counts (percentages), respectively

CRRT – continuous renal replacement therapy

FFP – fresh frozen plasma

IVIG – intravenous immunoglobulin

ECMO – extracorporeal membrane oxygenation

PICU – Pediatric intensive care unit

PARDS – Pediatric Acute Respiratory Distress Syndrome

**eTable 4: Cause of death in patients with PARDS**

| **Cause of death** | **Mild PARDS**  **(n=7)** | **Moderate PARDS (n=4)** | **Severe PARDS (n=10)** |
| --- | --- | --- | --- |
| Refractory hypoxemia |  |  | Pneumonia (6) |
| Multiorgan failure | Sepsis (2) | Sepsis (3) | Sepsis (2) |
| Poor overall prognosis | Acute leukemia (1)  Hemophagocytic lymphohistiocytosis (1) |  |  |
| Poor neurologic prognosis | Cardiac arrest (1)  Drowning (1)  Brainstem encephalitis (1) | End-stage cystic  encephalomalacia (1) | Cardiac arrest (2) |

PARDS – Pediatric Acute Respiratory Distress Syndrome

**eFigure 1: Ventilation parameters in patients with PARDS across 28 days**


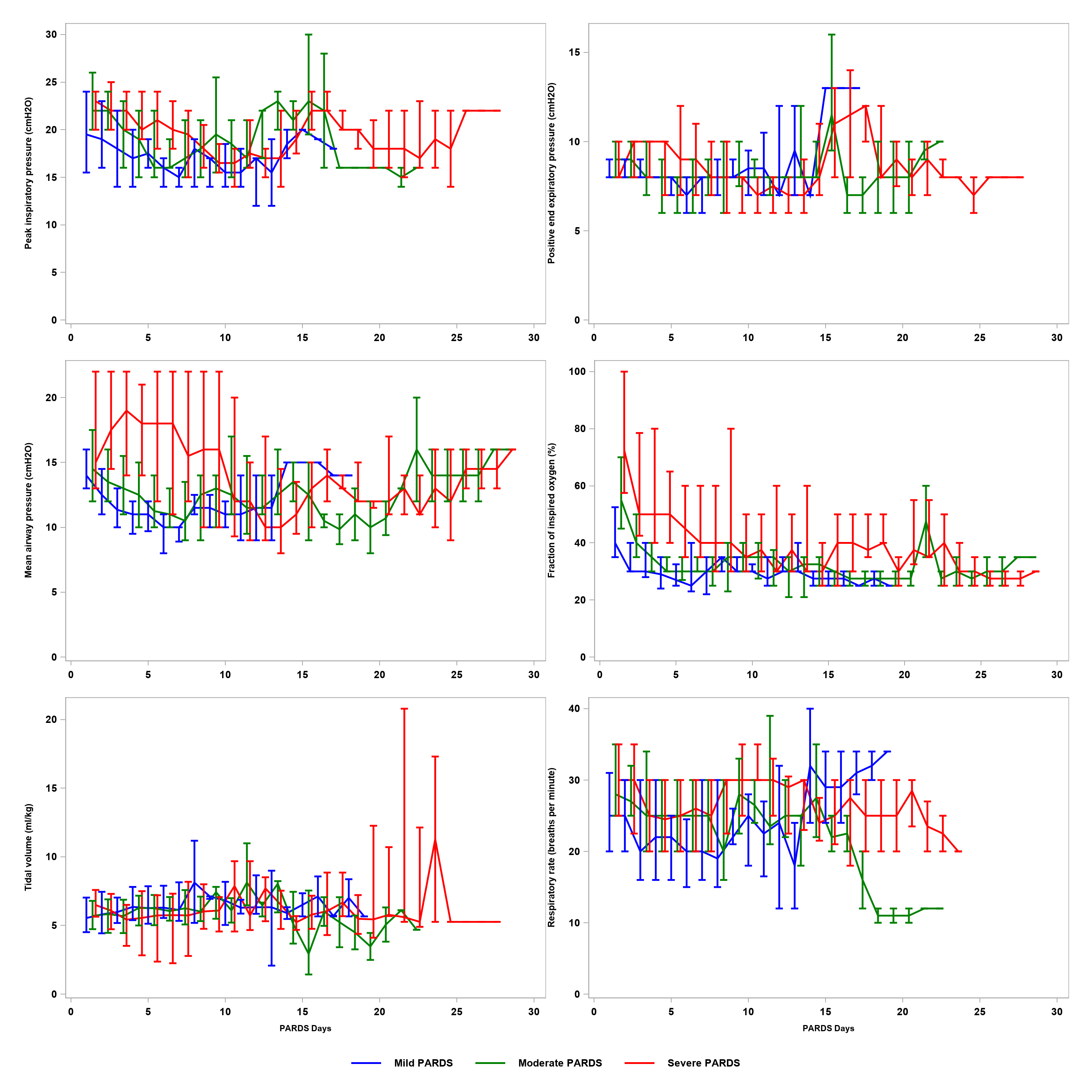


PARDS – Pediatric Acute Respiratory Distress Syndrome

**eFigure 2: Kaplan-Meier curves of duration of mechanical ventilation in patients with mild, moderate and severe PARDS**


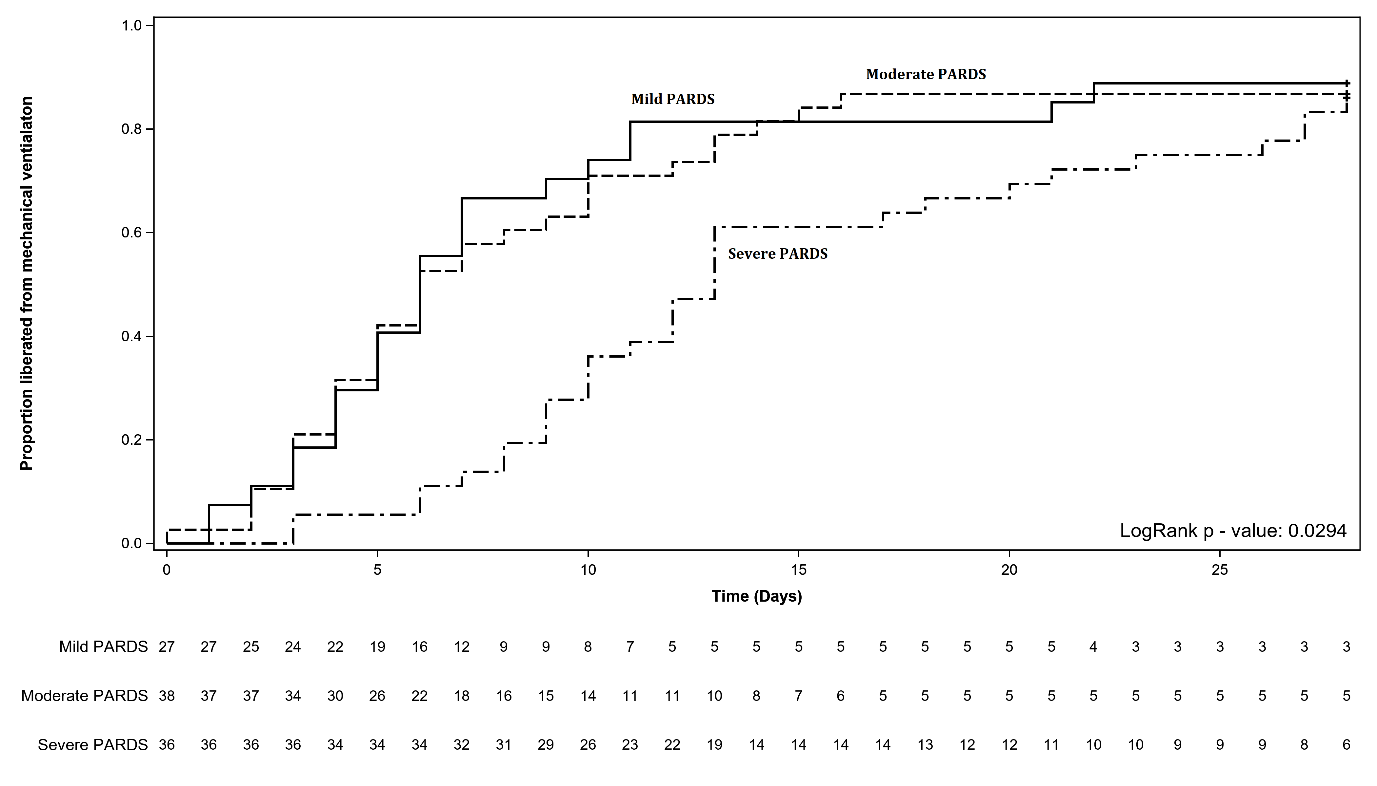


PARDS – Pediatric Acute Respiratory Distress Syndrome

**eFigure 3: Kaplan-Meier curves of PICU length of stay in patients with mild, moderate and severe PARDS**


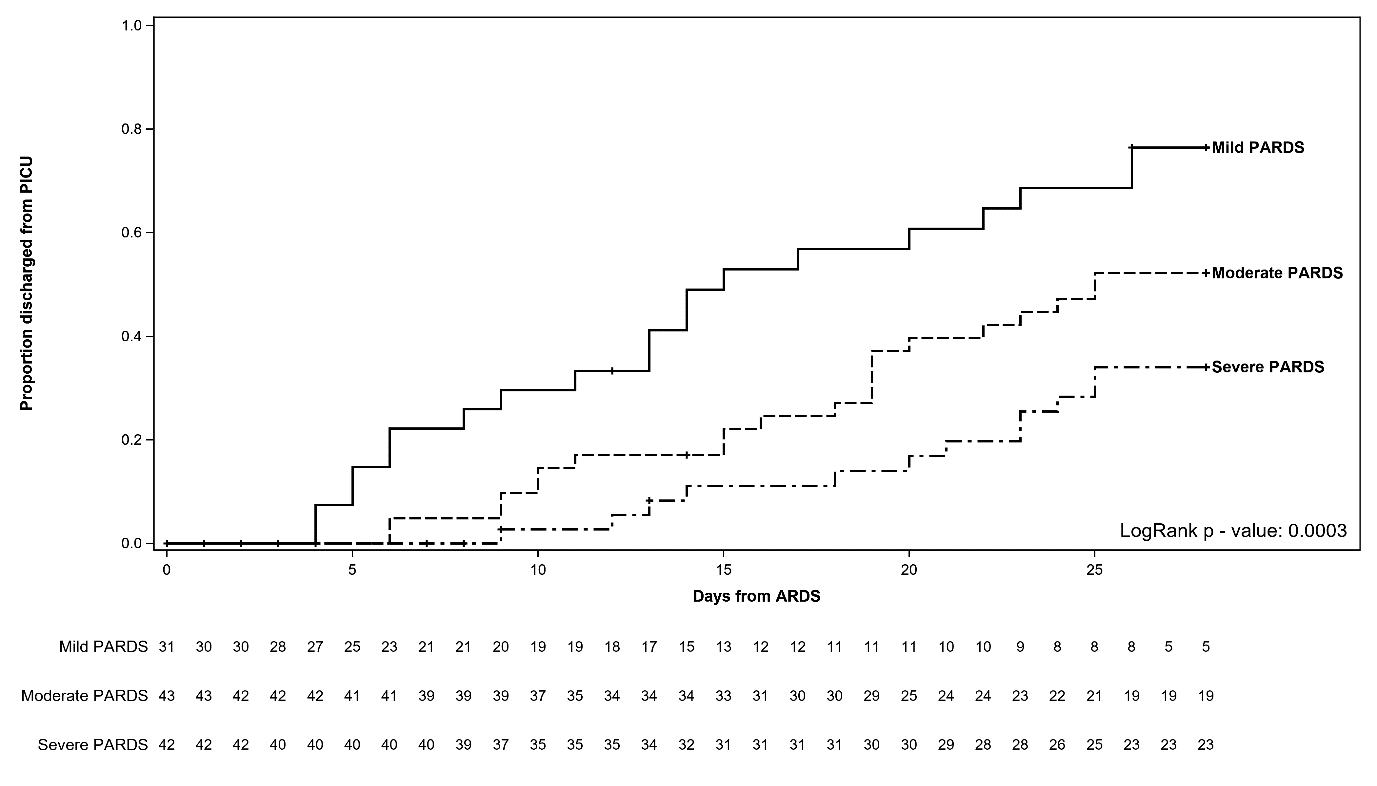


PICU – Pediatric intensive care unit

PARDS – Pediatric Acute Respiratory Distress Syndrome

**eFigure 4: Kaplan-Meier curves of hospital length of stay in patients with mild, moderate and severe PARDS**


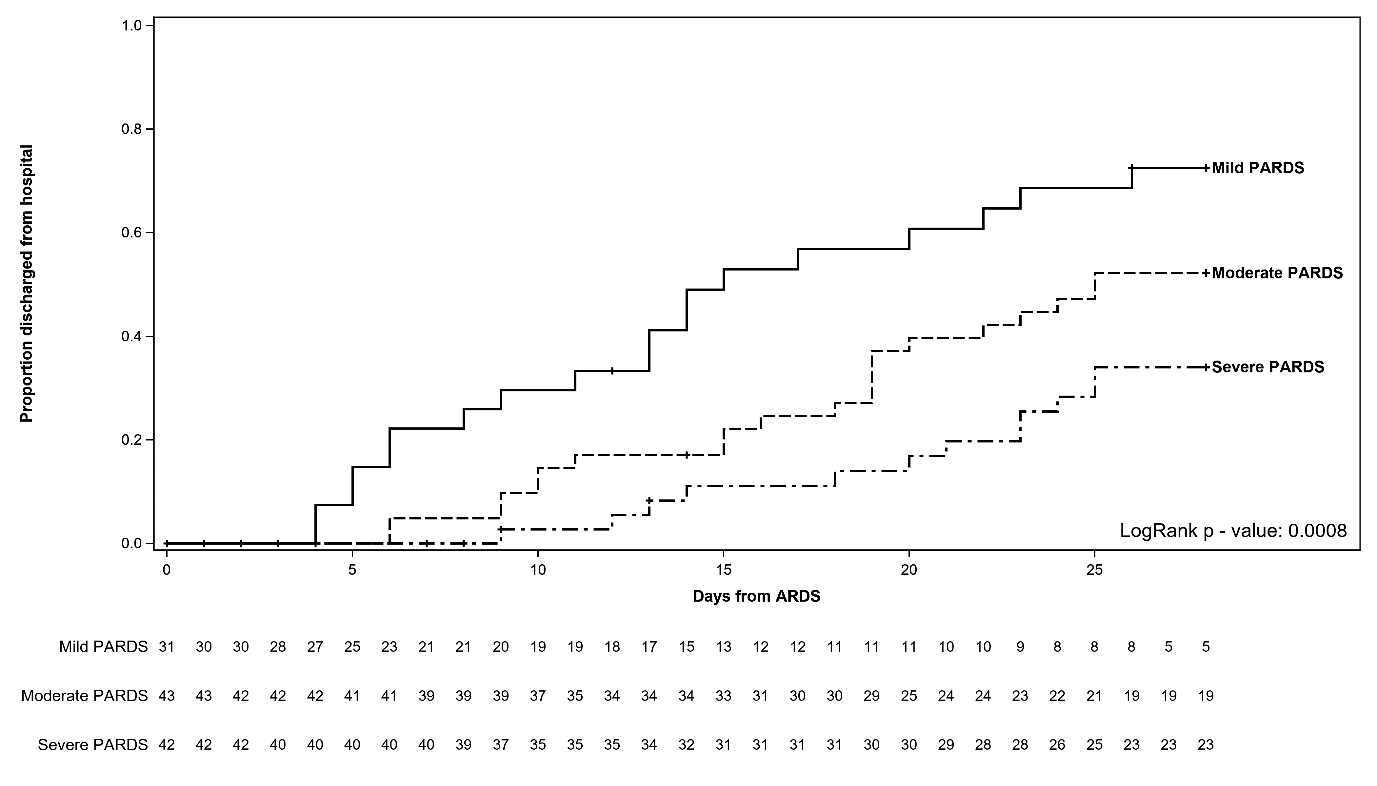


PARDS – Pediatric Acute Respiratory Distress Syndrome

**eFigure 5: Kaplan-Meier curve for outcome of PICU mortality in patients with mild, moderate and severe PARDS (top) and sensitivity analysis (bottom) excluding patients who at PICU admission, had a poor overall or poor neurologic prognosis**

**
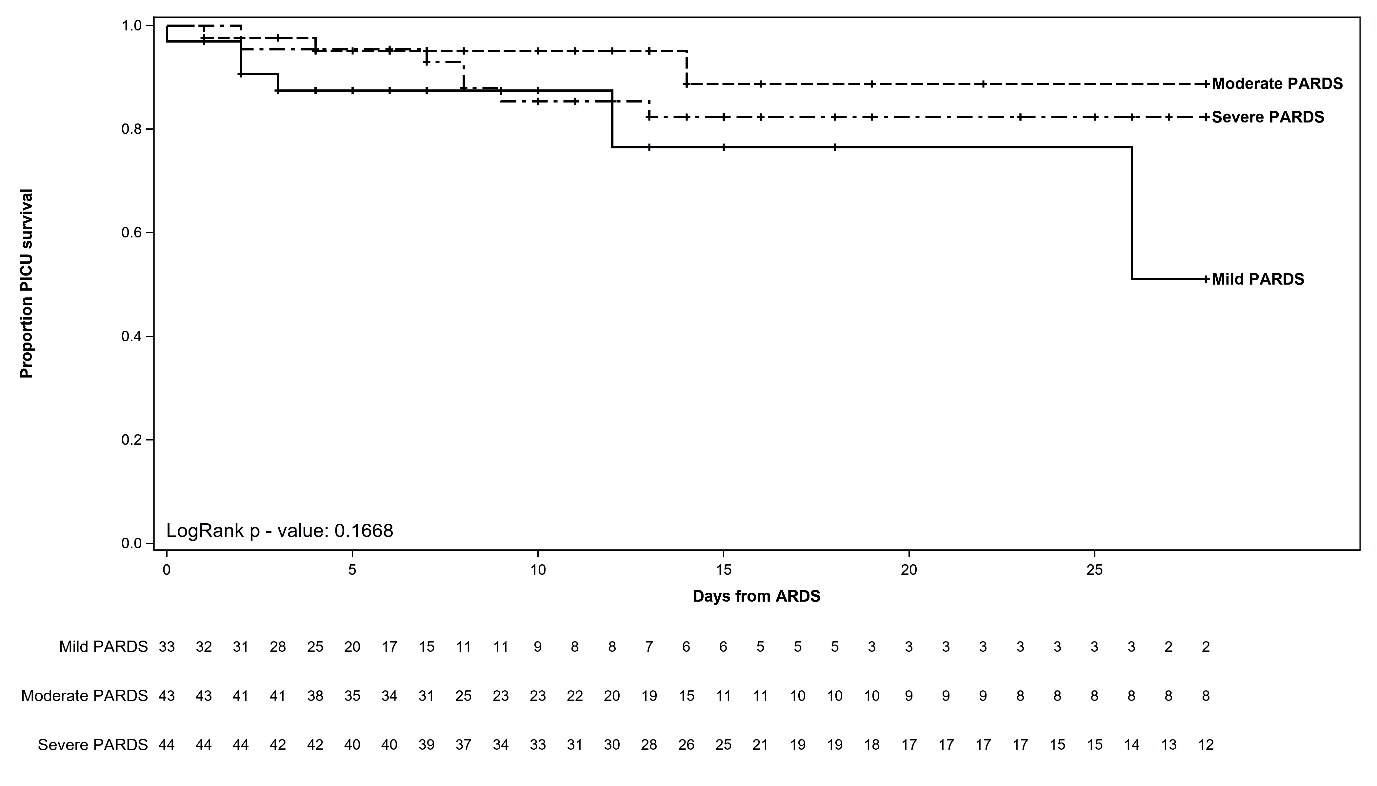
**


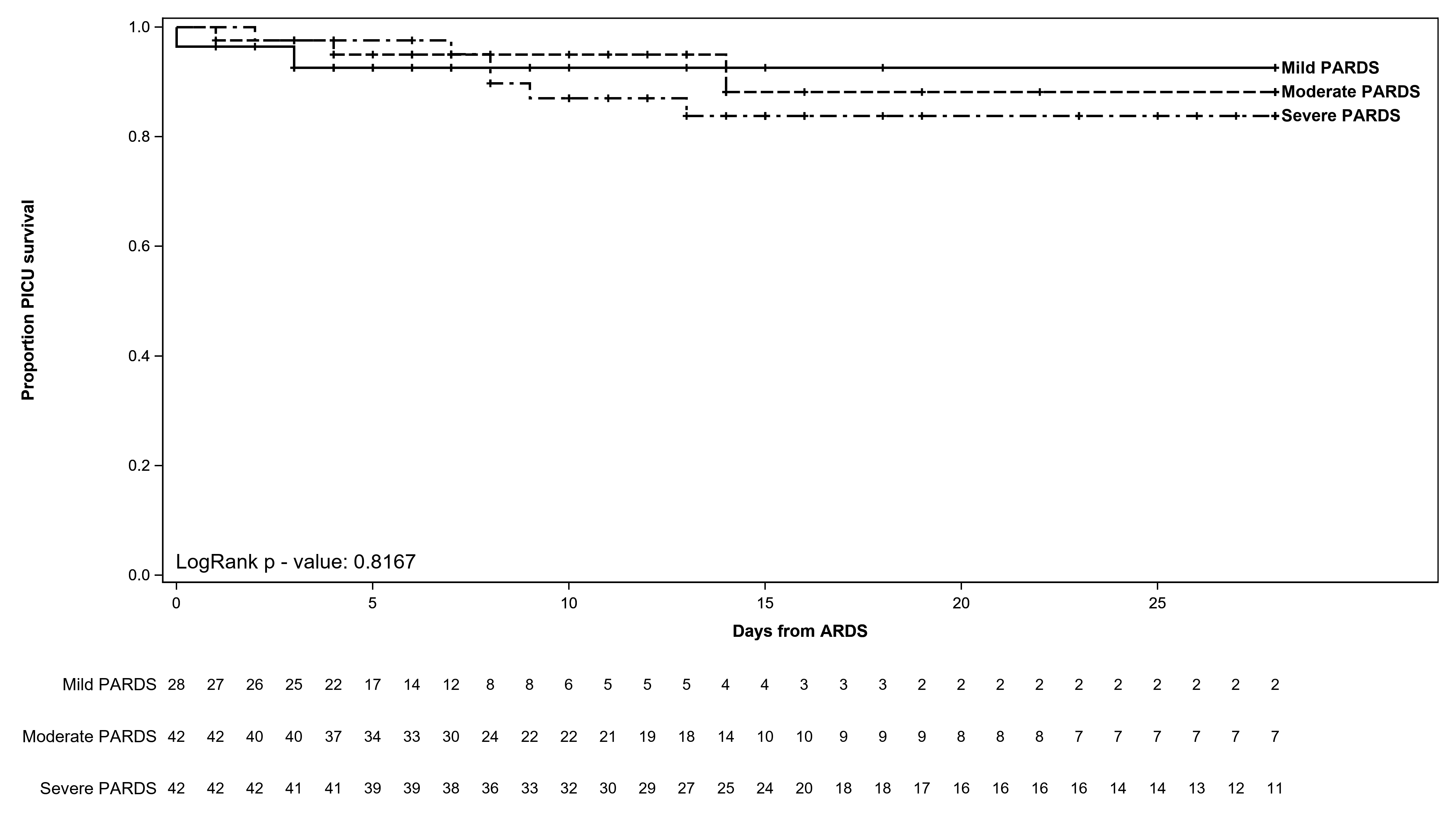


PICU – Pediatric intensive care unit

PARDS – Pediatric Acute Respiratory Distress Syndrome

**eFigure 6: Kaplan-Meier curve for outcome of hospital mortality in patients with mild, moderate and severe PARDS (top) and sensitivity analysis (bottom) excluding patients who at PICU admission, had a poor overall or poor neurologic prognosis**


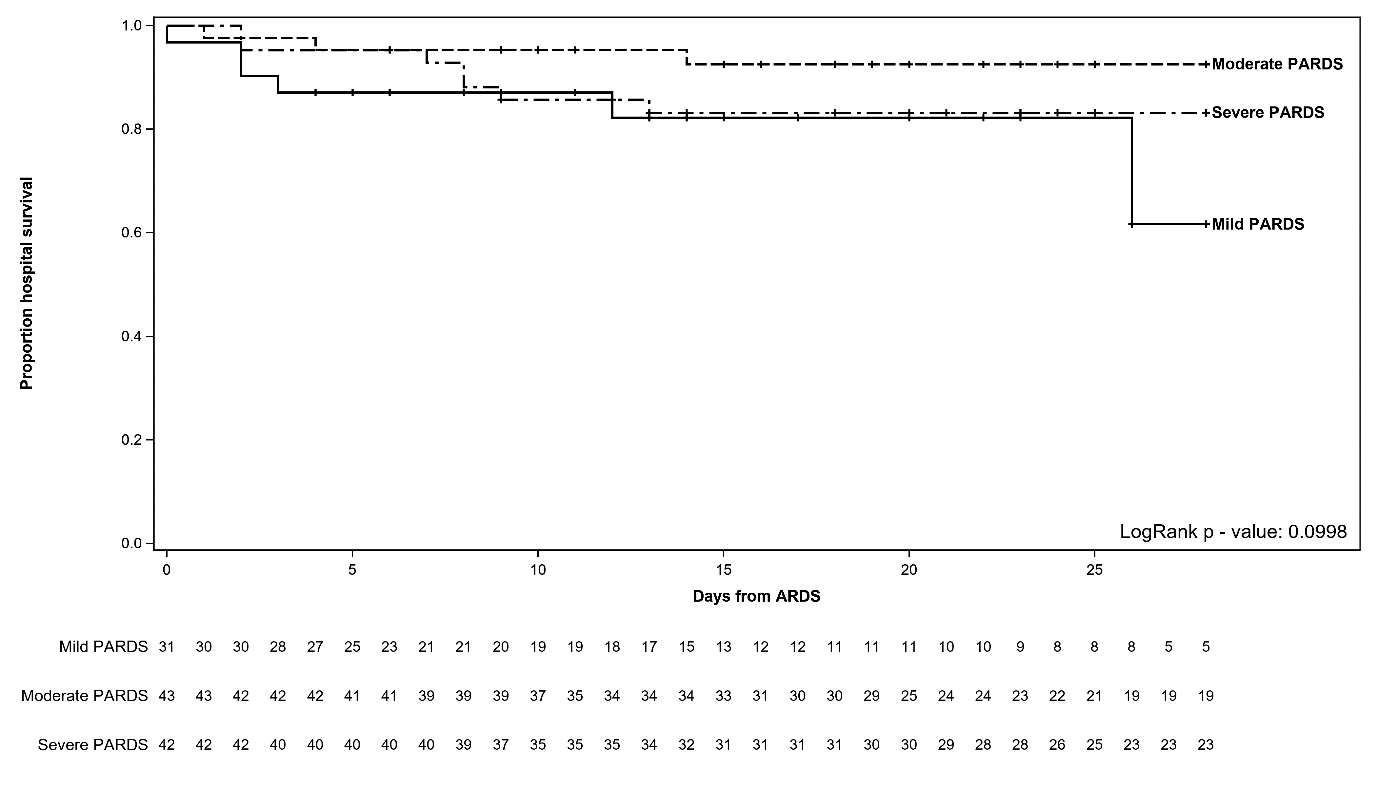


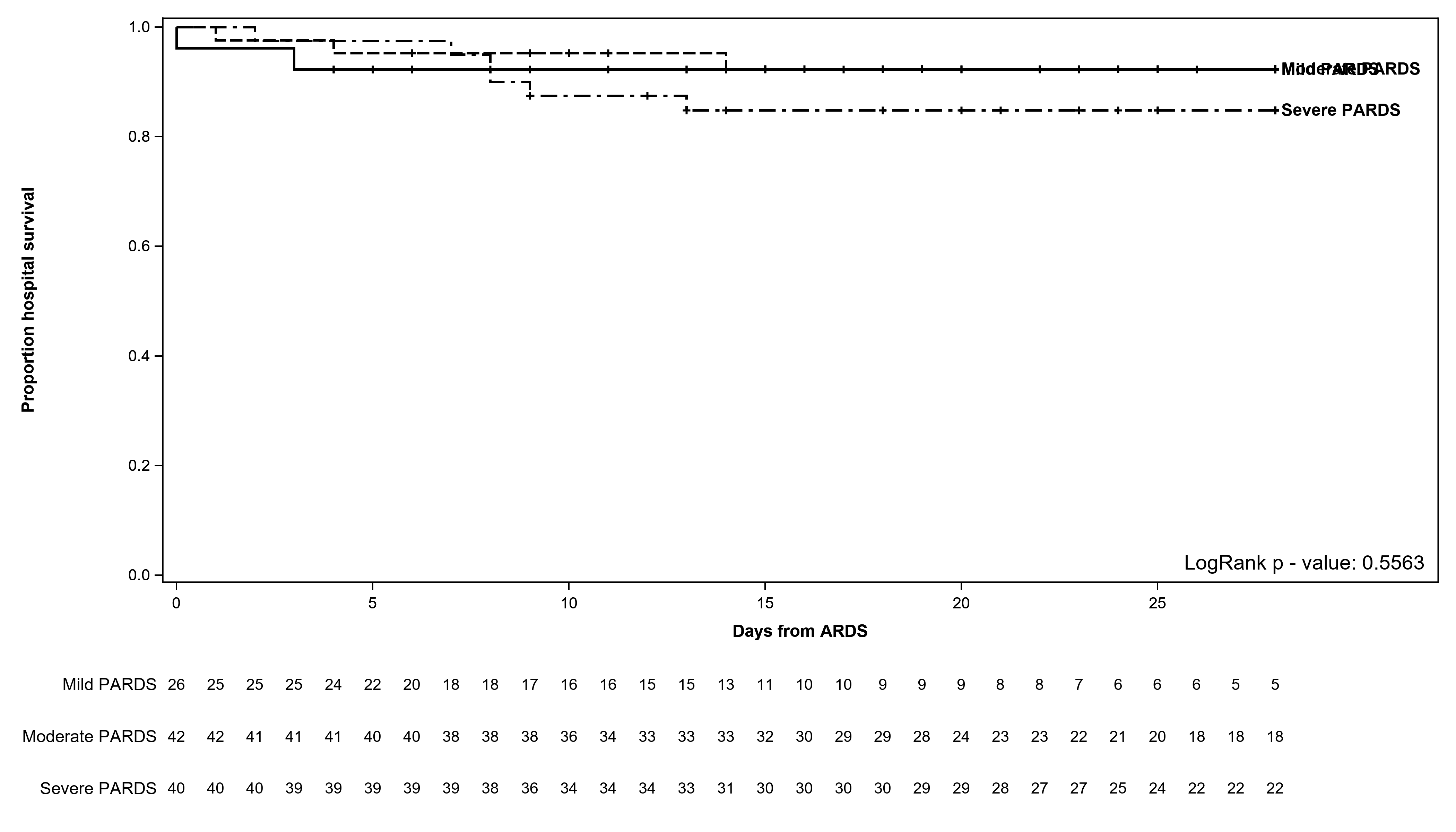


PARDS – Pediatric Acute Respiratory Distress Syndrome

**eFigure 7: Timeline of time to resolution of PARDS and discharge from the intensive care unit**


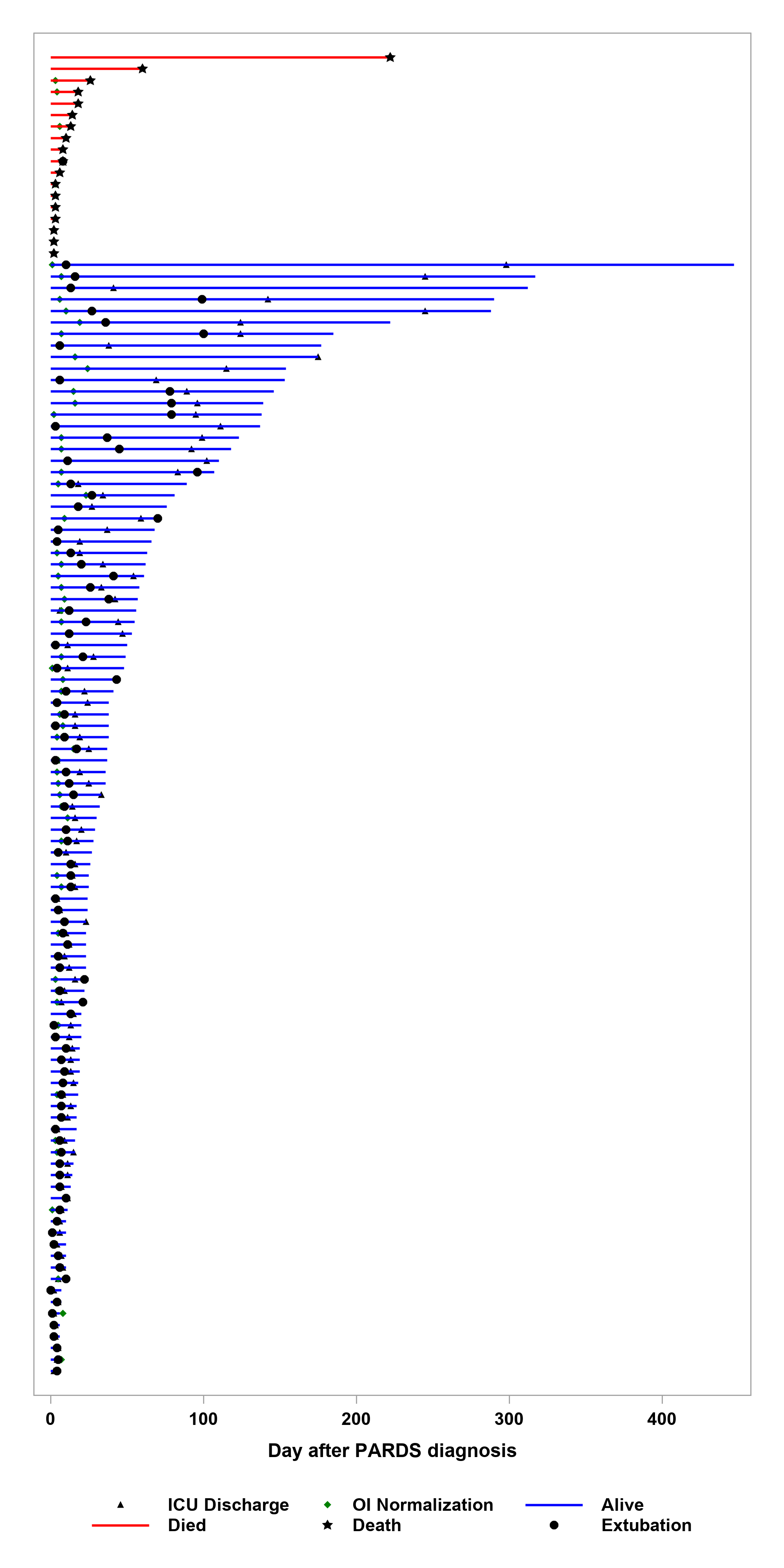


Each bar represents the course of PARDS in the ICU
